# Prognostic factors in aneurysmal subarachnoid hemorrhage during the clazosentan era: a multicenter study using multivariate analyses and machine learning model

**DOI:** 10.1007/s10143-025-03858-7

**Published:** 2025-10-10

**Authors:** Shinsuke Muraoka, Takashi Izumi, Kazuki Ishii, Issei Takeuchi, Masahiro Nishihori, Shunsaku Goto, Fumiaki Kanamori, Kenji Uda, Yusuke Sakamoto, Kinya Yokoyama, Kazuki Nishida, Basile Chretien, Ryuta Saito

**Affiliations:** 1 Department of Neurosurgery, Nagoya University Graduate School of Medicine, Tsurumai-cho 65, Showa-ku, Nagoya, Aichi Japan; 2 Department of Neurosurgery, Nagoya Ekisaikai Hospital, Nagoya, Aichi Japan; 3 Department of Neurosurgery, Japanese Red Cross Aichi Medical Center Nagoya Daini Hospital, Nagoya, Aichi Japan; 4 Department of Neurosurgery, Japan Community Health Care Organization Chukyo Hospital, Nagoya, Aichi Japan; 5 Department of Biostatistics, Kyoto University School of Public Health, Kyoto, Japan; 6 Department of Advanced Medicine, Nagoya University Hospital, Nagoya, Aichi Japan

**Keywords:** Aneurysm, Subarachnoid hemorrhage, Prognostic factors, Outcomes, Machine learning, SHapley additive explanations analysis

## Abstract

**Background:**

Controlling delayed cerebral ischemia (DCI) is crucial for improving the prognosis in patients with aneurysmal subarachnoid hemorrhage (aSAH). This study aimed to identify prognostic factors in patients with aSAH in Japan.

**Methods:**

A multicenter, retrospective, observational cohort study was conducted from April 2021 to March 2024. Patients underwent surgical repair of ruptured aneurysms within 48 h of onset, followed by postoperative treatment with multiple drugs, including clazosentan and fasudil, to prevent cerebral vasospasm. The primary outcome was the proportion of patients with a good outcome, defined as a modified Rankin Scale score of 0 to 2 at discharge. Multivariate logistic regression and stepwise model selection were applied to identify prognostic factors. SHapley Additive exPlanations (SHAP) analysis was used to visualize the relative importance of predictors and their impact on outcomes.

**Results:**

Among 506 patients (mean age 63.5 years, 66.6% female), 53.0% achieved a favorable outcome. In multivariate analysis, treatment with clazosentan was associated with 1.84 times higher odds of a favorable outcome (*p* = 0.021), increasing to 1.97 when clazosentan was administered without fluid retention complications (*p* = 0.010). SHAP analysis further highlighted the impact of each factor on prognosis, identifying a lower WFNS grade, lower Fisher grade, younger age, clazosentan, cilostazol, and statin use as significant predictors of favorable outcomes.

**Conclusion:**

Clinical status at aSAH onset and age are uncontrollable factors; therefore, improving prognosis requires targeted prevention of DCI and effective management of brain edema. The administration of therapies such as clazosentan, cilostazol, and statins may contribute to favorable outcomes.

**Clinical trial number:**

Not applicable

**Supplementary Information:**

The online version contains supplementary material available at 10.1007/s10143-025-03858-7.

## Introduction

According to a large-scale systematic review by Etminan et al., the global average incidence of aneurysmal subarachnoid hemorrhage (aSAH) is approximately 6–9 cases per 100,000 people per year, and the annual incidence of aSAH varies depending on regional and racial differences, smoking prevalence, and hypertension control [9]. In Japan, as in other countries, the number of aSAH cases is influenced by societal aging and lifestyle changes. Although the mortality rate associated with aSAH has shown some improvement, approximately 15–30% of patients die during the acute phase, and many survivors experience long-term disabilities. Mortality from rebleeding or brain edema during the acute phase remains high, with reports indicating that approximately 10% of patients die within 24 h and 20–30% within 1 month [6, 14, 25, 36].

Owing to advancements in specialized team-based approaches and improvements in medical systems led by neurosurgeons and emergency physicians, the acute-phase mortality rate has decreased. However, approximately half of survivors suffer from sequelae, such as motor impairments or cognitive dysfunction. The proportion of patients achieving a favorable functional prognosis (modified Rankin Scale [mRS] 0–2) varies greatly, depending on the neurological grade at admission and the presence of complications [37]. Older patients and severe cases often struggle to maintain independence in the activities of daily living over the long term.

For the treatment of delayed cerebral ischemia (DCI) following aSAH, the primary approach has traditionally focused on suppressing vasospasm or increasing cerebral blood flow. In recent years, however, multiple drugs have been investigated and applied to address vasospasm and other contributing factors, such as inflammation, microcirculation impairment, microthrombosis, and cortical spreading depolarization. A recently published network meta-analysis demonstrated the efficacy of myriad drugs in the prevention and treatment of DCI [7]. Nimodipine remains the most established drug therapy for the prevention of DCI after aSAH [24]. In Japan, where nimodipine is not available, alternative drugs such as fasudil, cilostazol, and statins are commonly used in combination to prevent cerebral vasospasm. In 2022, the endothelin A receptor antagonist clazosentan became available in Japan, although as of 2024, its use was limited to clinical settings within the country [8, 12, 28–30, 33].

Identifying and verifying risk factors from large medical datasets is often a time-consuming process. However, machine learning methods can efficiently identify factors strongly associated with outcomes, such as stroke onset, by analyzing a large number of features. In recent years, machine learning has improved the accuracy of stroke risk prediction and helped identify novel risk factors [4]. However, interpreting the results of machine learning and deep learning models remains a significant challenge. While machine learning has been applied successfully to cancer diagnosis, detection, prediction, and prognosis, it is often treated as a “black box,” limiting the interpretability of its predictions for patients and clinicians [2]. To address this limitation, Lundberg et al. proposed the use of SHapley Additive exPlanations (SHAP), a method based on game theory, to explain machine learning predictions [20]. Since its introduction, explainable machine learning models using SHAP have been applied to predict prognoses in various medical fields, including oncology and cardiovascular disease [1, 31].

This study aimed to investigate the prognostic factors in patients with aSAH in Japan, where various drugs are available, and to evaluate the impact of each factor on patient outcomes using SHAP.

## Materials and methods

This multicenter, retrospective, observational cohort study used data from patients with aSAH to investigate prognostic factors. This study was conducted in accordance with the 1964 Declaration of Helsinki and its later amendments and approved by the Institutional Review Board (IRB) of Nagoya University Hospital (approval number: 2022 − 0476). Informed consent was waived as the analysis used anonymized clinical data collected after obtaining written informed consent for treatment. Additionally, patient approval was obtained via an IRB-approved opt-out poster displayed in the outpatient department and neurosurgery ward.

### Study design and population

This retrospective study included all patients with subarachnoid hemorrhage who underwent definitive surgery for ruptured aneurysms between April 2021 and March 2024, followed by postoperative treatment with fasudil and/or clazosentan. The diagnosis of aSAH was based on the American Heart Association/American Stroke Association guidelines [14], using head computed tomography (CT), CT angiography, or digital subtraction angiography. The study included patients who underwent definitive surgery for ruptured cerebral aneurysms within 48 h of onset and received postoperative treatment to prevent cerebral vasospasm. Only patients who were independent in their daily activities prior to symptom onset were included. The exclusion criteria were: (1) incomplete data (e.g., missing mRS scores at discharge or perioperative complications), (2) no administration of clazosentan or fasudil, and (3) re-rupture during the hospitalization.

### Clinical data collection

Data were recorded on an electronic sheet, including age, sex, body mass index, comorbidities (hypertension, diabetes, dyslipidemia), smoking history, mRS score before onset and at discharge, World Federation of Neurosurgical Societies (WFNS) grade, Fisher grade, aneurysm location, surgical procedure, cerebrospinal fluid (CSF) drainage, postoperative medications, angiographic cerebral vasospasm, early brain injury, vasospasm-related DCI, other causes of cerebral infarction, and postoperative complications (brain edema, hypotension, pulmonary complications, and de novo intracranial hemorrhage [ICH]). DCI was defined as the appearance of a new high-intensity area on MRI, occurring between 4 and 20 days post-aSAH, indicating recent cerebral infarction that was not present on initial and immediate postoperative CT scans. MRI, CT angiography, or digital subtraction angiography was used to confirm vasospasm as the cause of DCI, excluding other factors such as hypoxia, electrolyte imbalances, infection, hydrocephalus, or seizures. A ≥ 2-point decline in the Glasgow Coma Scale, with no evidence of treatment-related brain injury, was also required to define DCI. Early brain injury was defined as any brain parenchymal damage occurring within 72 h of onset. Cerebral infarctions attributed to surgical complications were categorized as other causes. Independent neurosurgeons or radiologists, blinded to the clinical course, reviewed the imaging studies. Angiographic cerebral vasospasms were assessed using MR angiography, with cerebral vasospasm defined as a ≥ 25% reduction in arterial diameter compared to preoperative CT angiography.

The “completion” of clazosentan therapy is defined as the full 14-day standard prophylactic administration period (standard dose in Japan: 10 mg/h) completed without early discontinuation or interruption due to adverse events or clinical deterioration.

### Outcome measures

The primary outcome was defined as a favorable outcome at discharge, as indicated by a binary mRS score of 0–2 (favorable) versus 3–6 (unfavorable). Secondary outcomes were binary variables indicating the presence or absence of postoperative complications (angiographic cerebral vasospasm, vasospasm-related DCI, brain edema, pulmonary complications, hypotension, and de novo ICH).

### Candidate risk factors

For the primary outcome analysis, candidate risk factors included the following: treatment initiated with clazosentan and/or fasudil, WFNS grade, age, Fisher grade, treatment procedure, CSF drainage, sex, body mass index (BMI), smoking status, hypertension, dyslipidemia, diabetes mellitus, ozagrel, aspirin, clopidogrel, cilostazol, statin, eicosapentaenoic acid (EPA).

The secondary outcome analyses included only baseline and treatment-related risk factors, excluding any outcomes as predictors. The candidate risk factors included clazosentan or fasudil administration, WFNS grade, age, Fisher grade, aneurysm location, treatment procedure, CSF drainage, sex, BMI, smoking status, hypertension, dyslipidemia, diabetes mellitus, ozagrel, aspirin, clopidogrel, cilostazol, statins, and EPA.

### Statistical analysis

The primary analysis aimed to identify the risk factors associated with prognosis in patients with aSAH, focusing on the association between initial drug administration (clazosentan vs. fasudil) and clinical outcomes. Patients were categorized according to their initial drug administration, either clazosentan, fasudil, or both. Logistic regression models were used to examine the associations between each risk factor and the primary outcome (favorable mRS score), as well as each secondary outcome. To select the most relevant risk factors, stepwise model selection using Akaike Information Criterion was performed. Forward and backward selections were applied, with cerebral vasospasm treatment as a mandatory covariate. The full model, comprising all candidate predictors, represented the upper limit, while the minimal model, consisting solely of cerebral vasospasm treatment, served as the lower limit. An additional logistic analysis was conducted to evaluate the impact of completing clazosentan treatment on the same outcomes as in the main analysis. Among patients treated with clazosentan, those who completed clazosentan were compared to those who discontinued clazosentan and instead started treatment with fasudil. A similar stepwise variable selection procedure was used in these analyses, with the scope restricted to models that included completion of clazosentan treatment as a mandatory covariate.

A supplementary analysis was conducted using random forest models to evaluate the relative importance of the candidate risk factors for each outcome. A model was constructed for each outcome using all candidate risk factors as explanatory variables. SHAP was applied to visualize the influence distribution for each risk factor, and the top 15 risk factors for each outcome were presented as SHAP score distributions.

All statistical analyses were performed using R version 4.4.1 (R Foundation for Statistical Computing, Vienna, Austria), with the significance level set at *p* < 0.05. Firth’s correction was applied to all the logistic regression models to reduce bias [10].

## Results

**Fig. 1:**
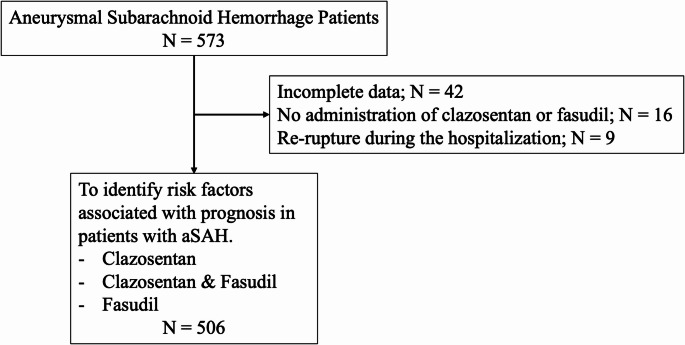
Flowchart of Patient Selection for Analysis of Aneurysmal Subarachnoid Hemorrhage This figure illustrates the selection process of patients with aneurysmal subarachnoid hemorrhage (aSAH), along with an overview of the analyses performed

A total of 506 patients (337 women, 66.6%; mean age 63.5 ± 13.8 years) were recruited for this study (Table 1). The median WFNS grade was 3 (interquartile range [IQR]: 2–4), and the median Fisher grade was 3. A total of 413 ruptured aneurysms (81.6%) were located in the anterior circulation. Of these patients, 213 (42.3%) underwent endovascular treatment and 272 (54.4%) underwent CSF drainage. Regarding perioperative drug administration, clazosentan was used in 193 cases (38.1%) and fasudil in 345 cases (68.3%). The oral medications included antiplatelet agents, EPA preparations, statins, and antiepileptic drugs. Among the antiplatelet agents, cilostazol was the most prescribed in 327 cases (64.9%). Statins were used in 271 cases (54.3%). The study flowchart is presented in Fig. 1.

**Table 1:** Patient baseline characteristics, postoperative used drugs, complications, and outcome

	Overall (*n* = 506)
Age, years, mean (SD)	63.5 ± 13.8
Female	337 (66.6)
BMI, kg/m^2^ median [IQR]	21.8 [19.6–24.8]
Comorbidities	
Hypertension	231 (45.8)
Diabetes mellitus	31 (6.1)
Dyslipidemia	90 (17.8)
Smoking	133 (27.1)
aSAH information	
WFNS grade, median [IQR]	3 [2–4]
I	91 (18.0)
II	150 (29.6)
III	60 (11.9)
IV	103 (20.3)
V	102 (20.2)
Fisher grade, median [IQR]	3 [3–3]
1	8 (1.6)
2	88 (17.4)
3	288 (56.9)
4	122 (24.1)
Aneurysm site (Anterior circulation)	413 (81.6)
Surgical information	
Endovascular treatment	213 (42.3)
CSF drainage	272 (54.4)
Perioperative used drugs	
Clazosentan	193 (38.1)
Fasudil	345 (68.3)
Ozagrel	320 (63.2)
Aspirin	30 (5.9)
Clopidgrel	11 (2.2)
Cilostazol	327 (64.9)
EPA	132 (26.1)
Statin	271 (54.3)
LEV	56 (11.1)
PER	21 (4.2)
Complication	
Early brain injury	66 (13.1)
Vasospasm (> 25%)	133 (26.3)
Vasospasm-related DCI	41 (8.1)
Date of vasospasm-related CI (POD), median [IQR]	9 [6–11]
Other cause CI	16 (3.3)
Brain edema	51 (10.2)
Pulmonary complication	55 (10.9)
Hypotension	13 (2.6)
De novo intracranial hemorrhage	15 (3.0)
Outcome	
Post mRS, median [IQR]	2 [1–4]
0	105 (20.8)
1	102 (20.2)
2	61 (12.1)
3	56 (11.1)
4	81 (16.0)
5	76 (15.0)
6	25 (4.9)
Favorable outcome (mRS 0–2)	268 (53.0)
Hospitalization period (days), median [IQR]	32 [22–47]

Values are n (%) unless otherwise stated

aSAH, aneurysmal subarachnoid hemorrhage; BMI, body mass index; CI, cerebral infarction; CSF, cerebrospinal fluid; DCI, delayed cerebral ischemia; EPA, eicosapentaenoic acid; IQR, interquartile range; LEV, levetiracetam; mRS, modified Rankin Scale; PER, perampanel; POD, postoperative day; SD, standard deviation; WFNS, World Federation of Neurosurgical Societies

### Primary outcomes

Early brain injury occurred in 66 cases (13.1%), and angiographic cerebral vasospasm was observed in 133 cases (26.3%). At discharge, the median mRS score was 2 (IQR: 1–4), with 268 patients (53.0%) achieving a favorable outcome (mRS 0–2). The median length of hospital stay was 32 days (IQR: 22–47).

**Fig. 2:**
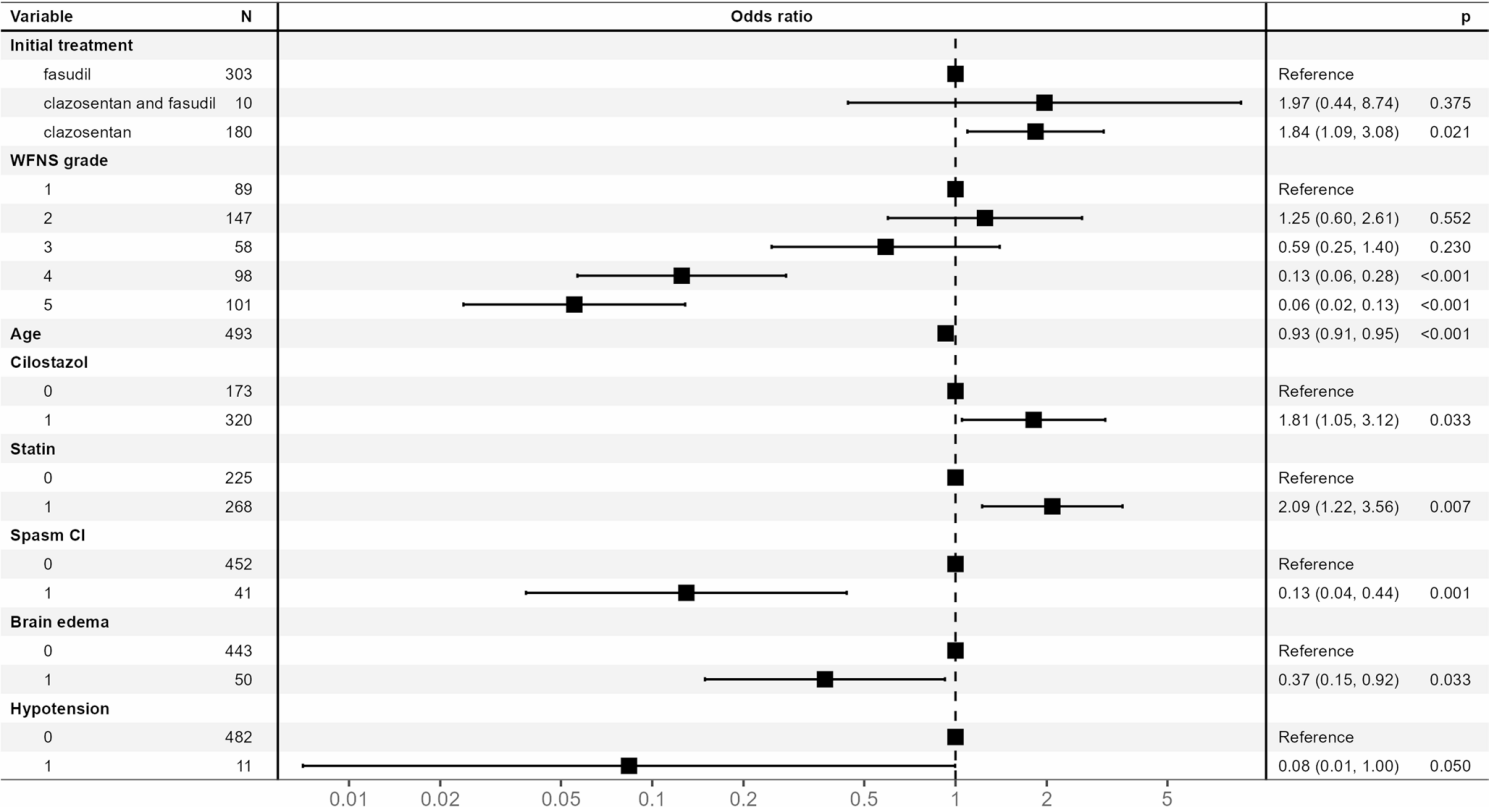
Forest Plot of Multivariate Logistic Regression Analysis Showing Associations with Favorable Outcomes This figure illustrates the odds ratio (OR) and 95% confidence interval (95% CI) for each factor, as estimated using multivariate logistic regression analysis. The horizontal axis represents OR on a logarithmic scale. Black squares indicate the point estimates for the OR, while horizontal lines represent the corresponding 95% CIs. The vertical dotted line represents an OR of 1. Factors with lines extending to the right of this line are associated with higher odds of favorable outcomes, whereas those extending to the left are associated with lower odds. The p values shown in the accompanying table indicate whether each factor has a statistically significant effect on the outcome. Statistical significance is set at *p* < 0.05. The “Variable” column lists the type and category of each factor analyzed, while the “N” column indicates the number of cases in each category. For example, under “Initial treatment,” the fasudil group serves as the reference category, and comparisons are made with the clazosentan monotherapy group and the fasudil combination group. For categorical variables, such as “WFNS grade,” the OR for other grades is compared against the OR for the reference (best) grade (e.g., grade 1). For continuous variables, such as “Age,” an OR < 1 indicates that the odds of a favorable outcome decrease as age increases

When focusing on differences in the initial drug type, multivariate analysis identified factors associated with favorable outcomes (mRS 0–2 at discharge; Fig. 2). Compared to fasudil, clazosentan increased the odds of a favorable outcome by 1.84 (95% confidence interval [CI]: 1.09–3.08; *p* = 0.021). Other factors that correlated with favorable outcomes included the administration of cilostazol and statins. Conversely, unfavorable outcomes were associated with WFNS grade IV and V, advanced age, vasospasm-related DCI, brain edema, and hypotension.

**Fig. 3:**
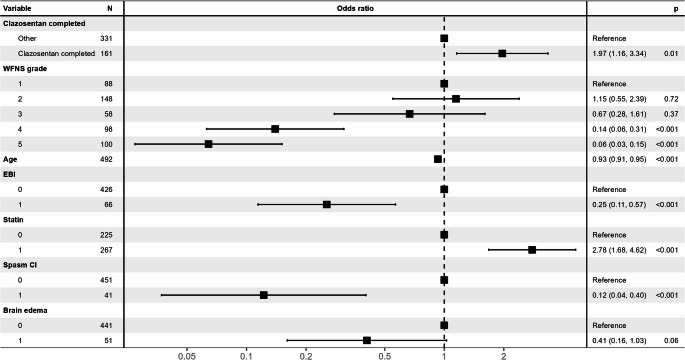
Multivariate Logistic Regression Analysis Results of the Relationship Between Clinical Factors and Outcomes (Forest Plot) This figure illustrates the results of a multivariate logistic regression analysis evaluating the relationship between clinical factors, including whether or not clazosentan treatment is completed (“Clazosentan completed” vs. “Other”), and outcomes in patients with aneurysmal subarachnoid hemorrhage (aSAH) caused by ruptured cerebral aneurysms. The horizontal axis represents the odds ratio (OR) on a logarithmic scale. Black squares indicate the point estimates of the OR, while horizontal lines represent the 95% confidence intervals (95% CI). The vertical dashed line (OR = 1) serves as a reference point, where values to the right indicate an increased likelihood of the outcome and values to the left indicate a decreased likelihood. The p-values shown in the table indicate statistical significance, with *p* < 0.05 considered statistically significant. For example, the group that has completed clazosentan treatment shows an OR of 1.97 (95% CI: 1.16–3.34, *p* = 0.01) compared to the reference group (“Other”), indicating a significant association with the outcome. The analysis also includes other variables, such as WFNS grade (reference: grade 1), age, early brain injury, use of statins, spasm CI, and brain edema. The odds ratio and 95% confidence interval (CI) are displayed for each variable, and corresponding p-values are used to assess the statistical significance and magnitude of each factor’s contribution to the outcome

Multivariate analysis, focusing on the completion of clazosentan administration, identified factors associated with a favorable outcome (mRS 0–2 at discharge; Fig. 3). The completion of clazosentan administration increased the odds of a favorable outcome by 1.97 (95%CI: 1.16–3.34; *p* = 0.010). Additional factors associated with favorable outcomes included WFNS grade I or II, younger age, statin use, as well as the absence of early brain injury, vasospasm-related DCI, and brain edema.

**Fig. 4:**
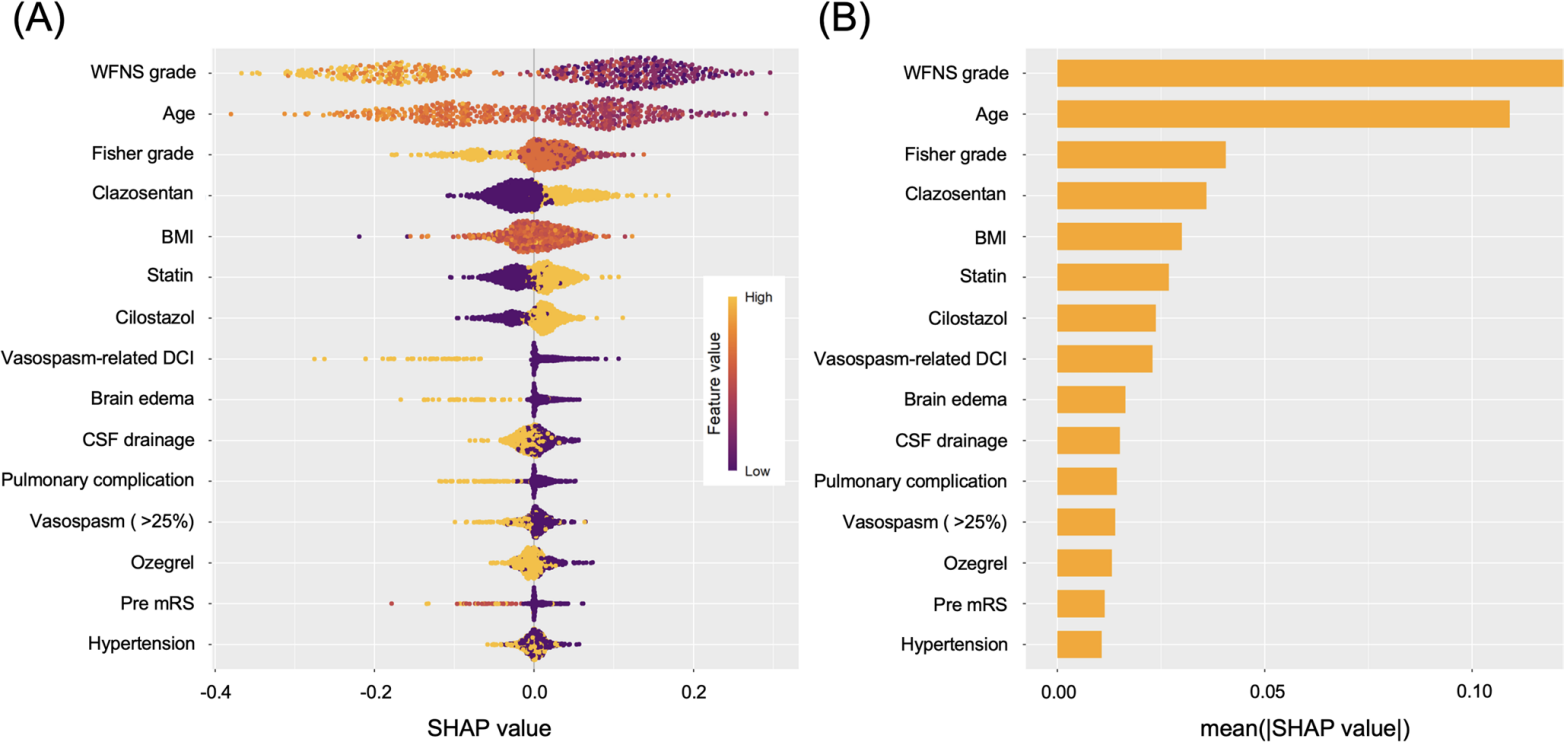
SHAP summary plot of favorable outcomes (**A**) This summary plot utilizes SHAP (Shapley Additive exPlanations) values to visualize the impact of each feature (factor) on prediction outcomes (e.g., good prognosis or poor prognosis) in the “subarachnoid hemorrhage (SAH) prognosis prediction” model built using machine learning. The features entered into the prediction model—such as age, grade, medication status, and clinical findings—are arranged vertically. Generally, features listed higher on the plot are more important to the model’s predictions, whereas those listed lower are less important. The horizontal axis represents SHAP values: features that extend predominantly to the right are “frequently associated with a good prognosis,” while those extending to the left are “frequently associated with a poor prognosis.” (**B**) This bar graph complements the SHAP summary plot shown above by presenting the “feature importance” based on the mean absolute SHAP values. In this graph, features are listed along the vertical axis, and the mean (|SHAP value|)—the average absolute SHAP value for each feature—is plotted on the horizontal axis. This representation highlights the overall contribution (importance) of each feature to the model’s predictions, allowing for a clearer understanding of their relative significance

According to SHAP analysis, the key factors influencing outcomes included WFNS grade; patient age; Fisher grade; administration of clazosentan, statins, or cilostazol; BMI; and the presence of vasospasm-related DCI. A favorable outcome was strongly associated with younger age, lower WFNS and Fisher grades, the absence of vasospasm-related DCI, and the use of clazosentan, statins, and cilostazol (Fig. 4).

### Secondary outcomes

As shown in Table 1, vasospasm-related DCI occurred in 41 cases (8.1%), with a median onset on postoperative day 9 (IQR: 6–11). Complications related to fluid retention included pleural effusion or pulmonary edema in 55 cases (10.9%) and brain edema in 51 cases (10.2%).

CSF drainage and ozagrel administration were associated with an increased risk of angiographic cerebral vasospasm, whereas endovascular treatment was associated with a reduction of this risk. Advanced age, CSF drainage, and clopidogrel use were identified as risk factors for vasospasm-related DCI, whereas endovascular treatment had a protective effect. While clazosentan did not significantly lower the incidence of angiographic cerebral vasospasm compared to fasudil, it significantly reduced the incidence of vasospasm-related DCI (Supplementary Fig. 1, Additional File 1). According to SHAP analysis, factors associated with the occurrence of angiographic vasospasm included CSF drainage, age, endovascular treatment of ruptured cerebral aneurysms, and the administration of clazosentan, cilostazol, or ozagrel (Supplementary Fig. 2, Additional File 1). In addition, SHAP analysis showed that the factors associated with the occurrence of vasospasm-related DCI included age, endovascular treatment of ruptured cerebral aneurysms, CSF drainage, WFNS grade, and clazosentan administration (Supplementary Fig. 3, Additional File 1).

No significant differences in fluid retention complications, including pleural effusion/pulmonary edema or brain edema, were observed between the clazosentan and fasudil groups (Supplementary Fig. 4, Additional File 1). Pulmonary complications were more common in patients with grade V WFNS and older patients. According to SHAP analysis, factors associated with the occurrence of pulmonary complications included the WFNS grade, age, Fisher grade, and endovascular treatment of ruptured cerebral aneurysms (Supplementary Fig. 5, Additional File 1). Higher WFNS grades (IV and V) were associated with an increased frequency of brain edema, whereas endovascular treatment was linked to a reduced incidence. The factors associated with the occurrence of brain edema, according to SHAP analysis, included WFNS grade, endovascular treatment of ruptured cerebral aneurysms, Fisher grade, and age (Supplementary Fig. 6, Additional File 1).

## Discussion

This study analyzed multiple factors influencing the prognosis of patients with aSAH and identified those associated with favorable clinical outcomes. More than half of the patients achieved good functional recovery after a median hospital stay of approximately one month. Consistent findings across both multivariate logistic regression and SHAP analysis highlighted the importance of WFNS grade, age, and clazosentan administration in predicting outcomes. In addition, cilostazol and statins were also associated with improved prognosis in the regression model. SHAP analysis further enhanced interpretability by illustrating the magnitude and direction of each variable’s effect at the individual patient level. Notably, Fisher grade emerged as more influential in SHAP rankings than in the regression results, suggesting potential nonlinear effects or interactions not fully captured by traditional statistical modeling. The complementary use of multivariate regression and explainable machine learning thus provides a more comprehensive understanding of prognostic factors and may help guide individualized treatment strategies.

### Outcomes in patients with subarachnoid hemorrhage

Data from the SAHIT (Subarachnoid Hemorrhage International Trialists) Repository [17] has shown that female sex, WFNS grades 4–5, cerebral vasospasm on imaging, worsening neurological symptoms, and DCI are associated with poor functional outcomes [11, 39]. Among these, cerebral infarction has a greater impact on outcomes than that of vasospasm [38]. The BEHAVIOR score serves as a predictive model for poor outcomes, incorporating factors such as Fisher grade ≥ 3 (1 point), age ≥ 55 years (1 point), Hunt/Hess grade ≥ 4 (1 point), ventriculostomy for acute hydrocephalus (1 point), vasospasm on initial angiography (3 points), intracranial pressure >20 mmHg (3 points), and treatment of multiple aneurysms (1 point) [16]. Previous studies have consistently identified advanced age as a significant predictor of poor outcomes and permanent neurological dysfunction in patients with SAH [27, 41].

Similar to previous reports, we found that severe cases, advanced age, cerebral vasospasm, and DCI were strongly associated with poor outcomes. Among these, severe cases and advanced age were particularly significant predictors of a poor prognosis. Although brain edema has not been extensively discussed in previous studies, our findings suggest that maintaining cerebral circulation and preventing cerebral vasospasm, alongside interventions such as ventricular drainage for acute hydrocephalus to control intracranial pressure, are critical for improving outcomes. Our current findings expand upon this by showing that pharmacologic interventions—specifically the administration of clazosentan, cilostazol, and statins—may also contribute to improved outcomes. Cilostazol has previously been linked to favorable outcomes in a network meta-analysis [7]. Moreover, in this study, the newly approved drug clazosentan, available since 2022 in Japan, also demonstrated a positive impact on patient outcomes—a finding of clinical significance.

Previous study showed the high incidence rate of fluid retention in clazosentan [8, 29, 30]. In this study, there was no significant difference in the overall incidence of fluid retention, such as pulmonary complications or cerebral edema, between the clazosentan and fasudil groups. This suggests that, although clazosentan carries a risk of fluid retention, appropriate fluid management may prevent the development of severe complications. Cerebral edema was identified as an important factor associated with poor outcomes. Although brain edema is more likely in patients with intracranial hematoma and may be exacerbated by clazosentan [28–30, 33], our study did not identify Fisher classification as a prognostic factor in the logistic regression analysis. However, SHAP analysis indicated a potential association between Fisher classification and clinical outcomes. While previous studies have reported a link between Fisher classification and prognosis, whether outcomes differ specifically in patients with Fisher grade 4 remains an important question for future research.

### Delayed cerebral ischemia

In this study, DCI was classified into three categories: early brain injury, DCI caused by cerebral vasospasm, and cerebral infarction due to other factors. DCI caused by cerebral vasospasm was associated with poor prognosis but occurred less frequently with clazosentan than with fasudil. Cerebral vasospasm involves the phosphorylation and dephosphorylation of myosin light chains through two primary pathways: a calcium-dependent calmodulin-mediated pathway and a calcium-independent Rho kinase-mediated pathway [34, 35]. In Japan, fasudil hydrochloride, a Rho kinase inhibitor, was the standard treatment for preventing cerebral vasospasm until 2021. In 2022, clazosentan, a selective endothelin A receptor antagonist, became available. Clazosentan inhibits the calcium-dependent calmodulin-mediated pathway and the calcium-independent Rho kinase-mediated pathway, making it a potent prophylactic agent against vasospasm [15, 32]. Clinical evidence suggests that clazosentan may improve functional outcomes in patients with aSAH. Cilostazol, which has a dual mechanism of action involving vasodilation through phosphodiesterase 3 inhibition and antiplatelet activity, also has a relatively high safety profile. Cilostazol has been suggested to improve neurological outcomes by inhibiting vasospasm after SAH [3, 5, 19], and its combination with standard therapies such as nimodipine is becoming increasingly supported by evidence. Other antiplatelet drugs, such as aspirin and clopidogrel, are frequently used to prevent thrombosis following stent-assisted aneurysm treatment. However, direct evidence supporting their efficacy in preventing vasospasms after SAH is limited. In this study, only cilostazol, which also exhibits vasodilatory effects, was associated with an improved prognosis. It is hypothesized that cilostazol not only prevents peripheral thrombosis but also improves microcirculation in the brain, contributing to better outcomes.

Nimodipine remains the cornerstone of global pharmacotherapy for preventing DCI after aSAH, with meta-analytic evidence showing a relative risk reduction of poor functional outcomes (RR = 0.69, 95% CI: 0.60–0.78), mortality (RR = 0.50, 95% CI: 0.32–0.78), and cerebral vasospasm (RR = 0.68, 95% CI: 0.46–0.99) compared to placebo or no treatment [13]. However, because nimodipine is not available in Japan, alternative agents such as fasudil, cilostazol, and clazosentan are used. In our study, clazosentan was associated with a 1.84-fold increase in the odds of favorable outcome, rising to 1.97 when treatment was completed without complications. Zhao et al. demonstrated that clinical outcomes were more favorable in the fasudil group than in the nimodipine group (*p* = 0.040) [40]. A Post-hoc propensity score-matched analysis of six randomized clinical trials found that clazosentan (10 mg/h) significantly reduced the incidence of cerebral vasospasm and associated morbidity and mortality following aSAH when compared to both placebo and nimodipine. The safety profiles were comparable across all groups [18].

While direct comparisons with nimodipine are not possible in this context, these effect sizes suggest that clazosentan may offer a clinically meaningful alternative. Further comparative effectiveness studies or network meta-analyses are needed to evaluate the relative efficacy of clazosentan within the global treatment landscape.

In randomized controlled trials conducted in Europe and the United States comparing clazosentan with placebo [21–23, 26], the 10 mg/h dose currently used in Japan was not evaluated. Moreover, in these studies, nimodipine was co-administered in over 90% of cases. As a result, the findings may not reflect a direct comparison between clazosentan and placebo, but rather suggest that clazosentan provides no additional benefit when used in combination with nimodipine.

The authors acknowledge some limitations in the present study. First, as a retrospective observational study, it inherently lacks control over exposure and outcome assessment. Prospective studies with long-term follow-up are required to assess the sustained impact of therapies such as clazosentan, cilostazol, and statins on functional outcomes and quality of life.

## Conclusions

In Japan, patients with aSAH typically remain hospitalized for approximately 1 month, with over half achieving favorable outcomes. Key factors linked to a better prognosis include endovascular treatment of ruptured aneurysms and the use of clazosentan, cilostazol, and statins, particularly the continued administration of clazosentan. Poor outcomes were associated with a high WFNS grade, older age, vasospasm-related DCI, and brain edema. Given that patient condition and age at SAH onset are uncontrollable factors, improving prognosis requires targeted DCI prevention and brain edema management.

aSAH, aneurysmal subarachnoid hemorrhage; BMI, body mass index; CI, cerebral infarction; CSF, cerebrospinal fluid; DCI, delayed cerebral ischemia; EPA, eicosapentaenoic acid; IQR, interquartile range; LEV, levetiracetam; mRS, modified Rankin Scale; PER, perampanel; POD, postoperative day; SD, standard deviation; WFNS, World Federation of Neurosurgical Societies.

## Supplementary Information

Below is the link to the electronic supplementary material.

Supplementary Material 1 (DOCX. 1.71 MB)

## Data Availability

The datasets used and/or analyzed during the current study are available from the corresponding author on reasonable request.

## Abbreviations

aSAH: aneurysmal subarachnoid hemorrhage
BMI: body mass index
CSF: cerebrospinal fluid
CT: computed tomography
DCI: delayed cerebral ischemia
EPA: eicosapentaenoic acid
ICH: intracranial hemorrhage
IRB: institutional Review Board
IQR: interquartile range
mRS: modified Rankin Scale
SHAP: SHapley Additive exPlanations
WFNS: World Federation of Neurosurgical Societies
