# Supplementary material for: Prognostic factors in aneurysmal subarachnoid hemorrhage during the clazosentan era: a multicenter study using multivariate analyses and machine learning model
Source: Neurosurg Rev. 2025 Oct 10;48(1):690. doi: 10.1007/s10143-025-03858-7 (PMC12513985; doi:10.1007/s10143-025-03858-7)
Supplement: Supplementary file 1 — Supplementary Material 1 (DOCX. 1.71 MB) [file 10143_2025_3858_MOESM1_ESM.docx]

SUPPLEMENTARY MATERIALS

**Title**: Prognostic Factors in Aneurysmal Subarachnoid Hemorrhage During the Clazosentan Era: A Multicenter Study Using Multivariate Analyses and Machine Learning

**Authors**: Shinsuke Muraoka, MD, PhD^1^*, Takashi Izumi, MD, PhD^1^, Kazuki Nishida, MD, PhD^2^, Basile Chretien, PharmD, MSc, MPH^2^, Kazuki Ishii, MD, PhD^1,3^, Issei Takeuchi, MD, PhD^1^, Masahiro Nishihori, MD, PhD^1^, Shunsaku Goto, MD, PhD^1^, Ryuta Saito, MD, PhD^1^

^1^ Department of Neurosurgery, Nagoya University Graduate School of Medicine, Nagoya, Aichi, Japan

^2^ Division of Biostatistics, Department of Advanced Medicine, Nagoya University Hospital, Nagoya, Aichi, Japan

^3^ Department of Neurosurgery, Nagoya Ekisaikai Hospital, Nagoya, Aichi, Japan

* **Corresponding author**

Shinsuke Muraoka, MD, PhD, MSc

Department of Neurosurgery, Nagoya University Graduate School of Medicine, Tsurumai-cho 65, Showa-ku, Nagoya, Aichi, Japan

E-mail: neuro-smuraoka@umin.ac.jp

ORCID: 0000-0003-4225-110X

**Supplementary Figure 1. Results of Multivariate Logistic Regression Analysis for Risk Factors Associated with Angiographic Vasospasm and Vasospasm-related DCI (Forest Plot)**


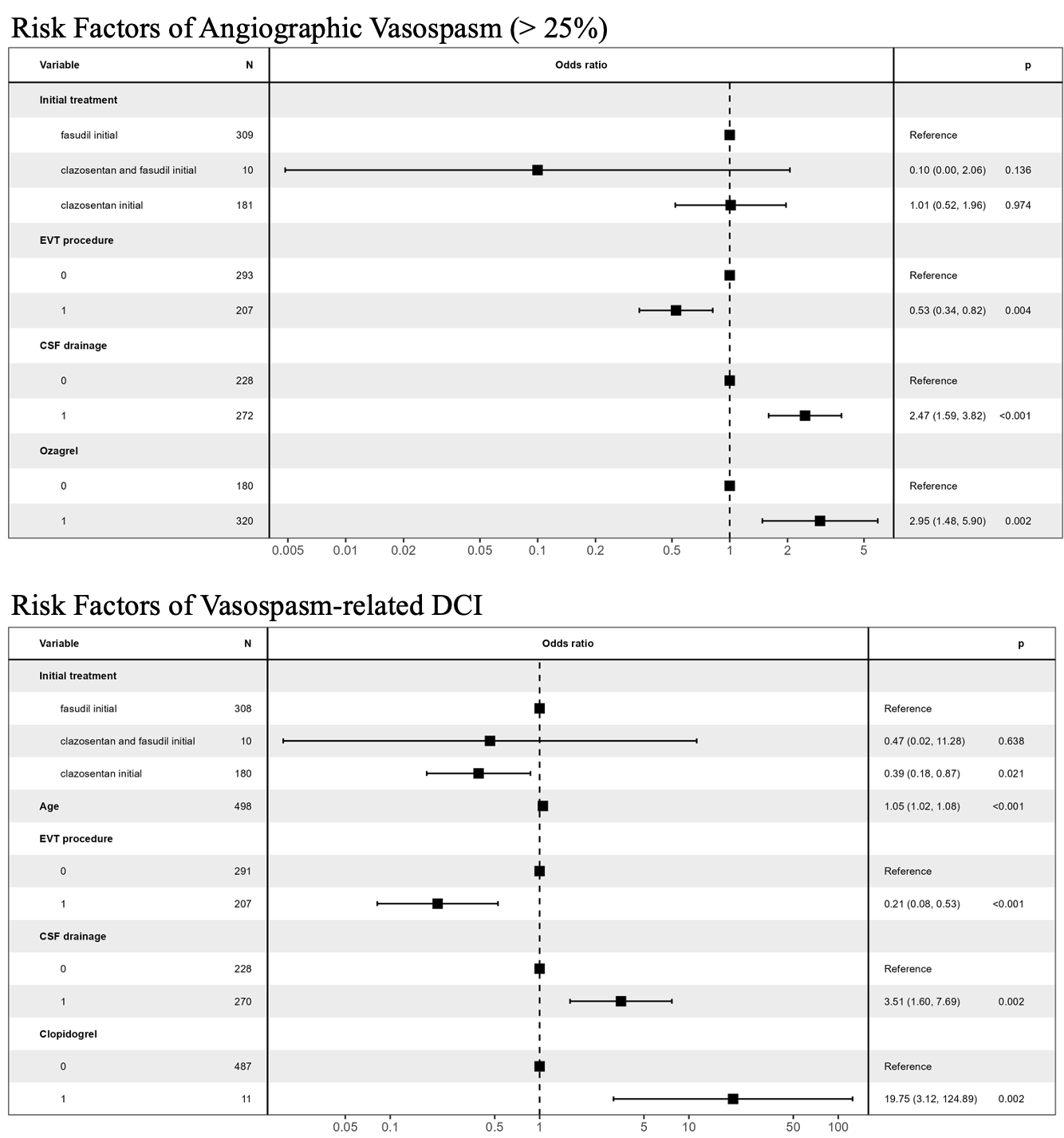


This figure presents the results of a multivariate logistic regression analysis evaluating risk factors for angiographic vasospasm (top panel) and vasospasm-related DCI (bottom panel) in patients with aneurysmal subarachnoid hemorrhage (aSAH). The horizontal axis represents the odds ratio (OR) on a logarithmic scale. Black squares indicate the point estimates of the OR, and horizontal lines represent the 95% confidence intervals (95% CI). The vertical dashed line (OR=1) serves as the reference point: values to the right indicate an increased risk, and values to the left indicate a decreased risk. P-values are displayed in the accompanying table, with p<0.05 considered statistically significant. In the top panel, which evaluates pulmonary complications, the explanatory variables included the type of initial treatment (reference: fasudil group), WFNS grade (reference: grade 1), age, and pre-mRS (modified Rankin Scale; reference: pre-mRS=0). The results show that older age, CSF drainage and clopidogrel administration were significantly associated with an increased risk of angiographic vasospasm. Endovascular treatment (EVT procedure) was significantly associated with an decreased risk of angiographic vasospasm. In the bottom panel, which evaluates vasospasm-related DCI, the same explanatory variables were used, with the presence or absence of brain edema as the outcome. The results suggest that CSF drainage and the administration of ozagrel were significantly associated with an increased risk of vasospasm-related DCI. Conversely, endovascular treatment was identified as a protective factor, significantly reducing the risk of vasospasm-related DCI.

**Supplementary Figure 2. Summary Plot Showing the Contribution of Each Variable (SHAP Value) in the Prediction Model: Angiographic Vasospasm**


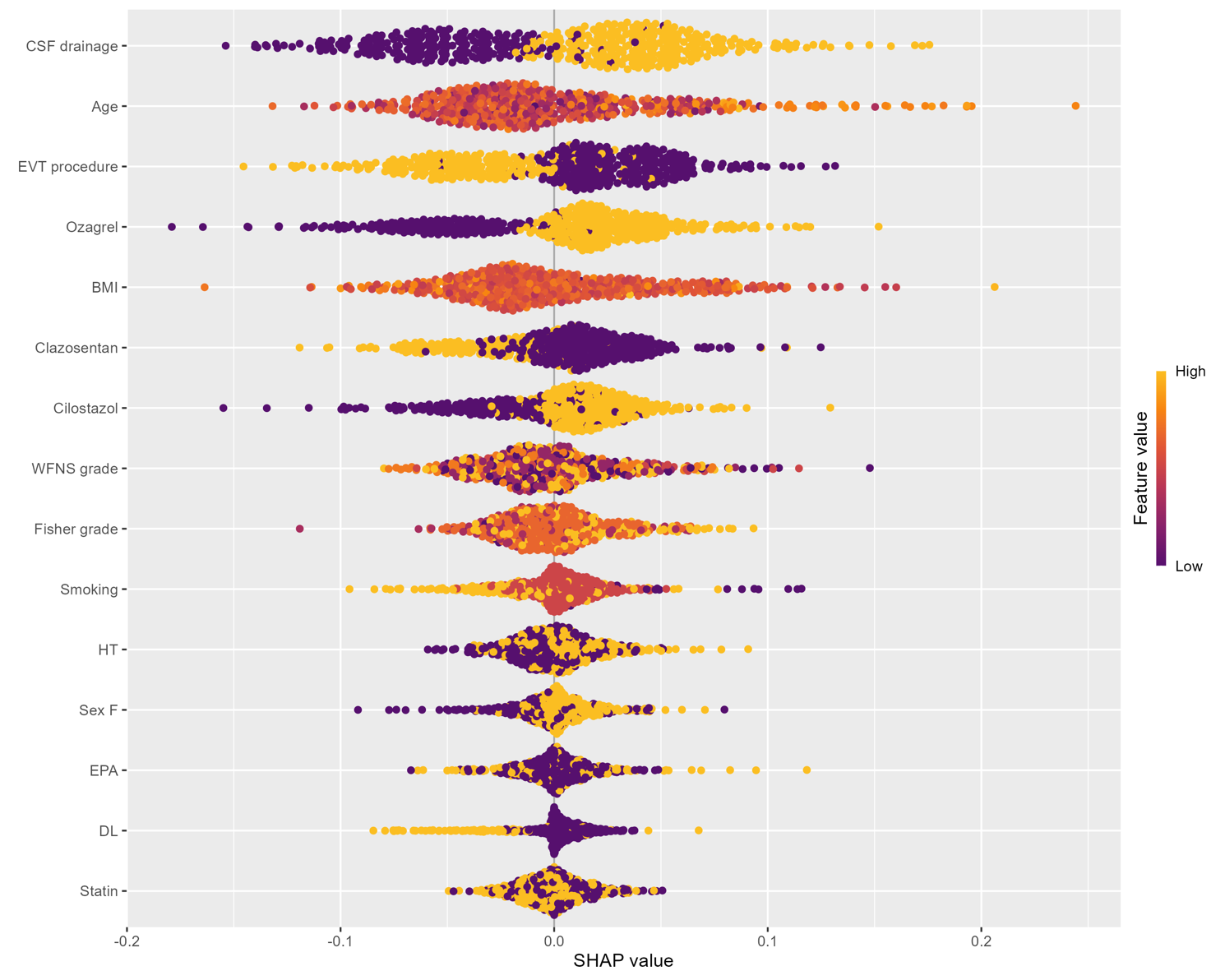


This figure presents a summary plot visualizing the contribution of each feature to the prediction of prognosis using SHAP (SHapley Additive exPlanations) values in a machine learning model. The vertical axis lists the features included in the model, including cerebrospinal fluid (CSF) drainage, age, endovascular treatment (EVT) procedure, ozagrel, body mass index (BMI), clazosentan, cilostazol, World Federation of Neurological Surgeons grade (WFNS) grade, Fisher grade, smoking, hypertension (HT), female sex (Sex F), Eicosapentaenoic acid (EPA), dyslipidemia (DL), and statin use. Each point on the vertical axis represents an individual case. The horizontal axis shows the SHAP values, which indicate the magnitude and direction of each feature’s contribution to the prediction. The color of the points reflects the value of the feature, where purple represents lower feature values and yellow represents higher feature values.

A positive SHAP value (points to the right of the vertical dashed line) indicates that the feature contributes to increasing the predicted outcome (e.g., higher risk of poor prognosis). Conversely, a negative SHAP value (points to the left) indicates that the feature contributes to decreasing the predicted outcome (e.g., lower risk of poor prognosis). Features are arranged on the vertical axis in descending order of their overall contribution to the model, allowing for a visual assessment of the importance of each factor. This plot enables intuitive understanding of both the magnitude and direction of each feature's impact on prediction, based on high and low values.

**Supplementary Figure 3. Summary Plot Showing the Contribution of Each Variable (SHAP Value) in the Prediction Model: Vasospasm-Related Delayed Cerebral Ischemia**


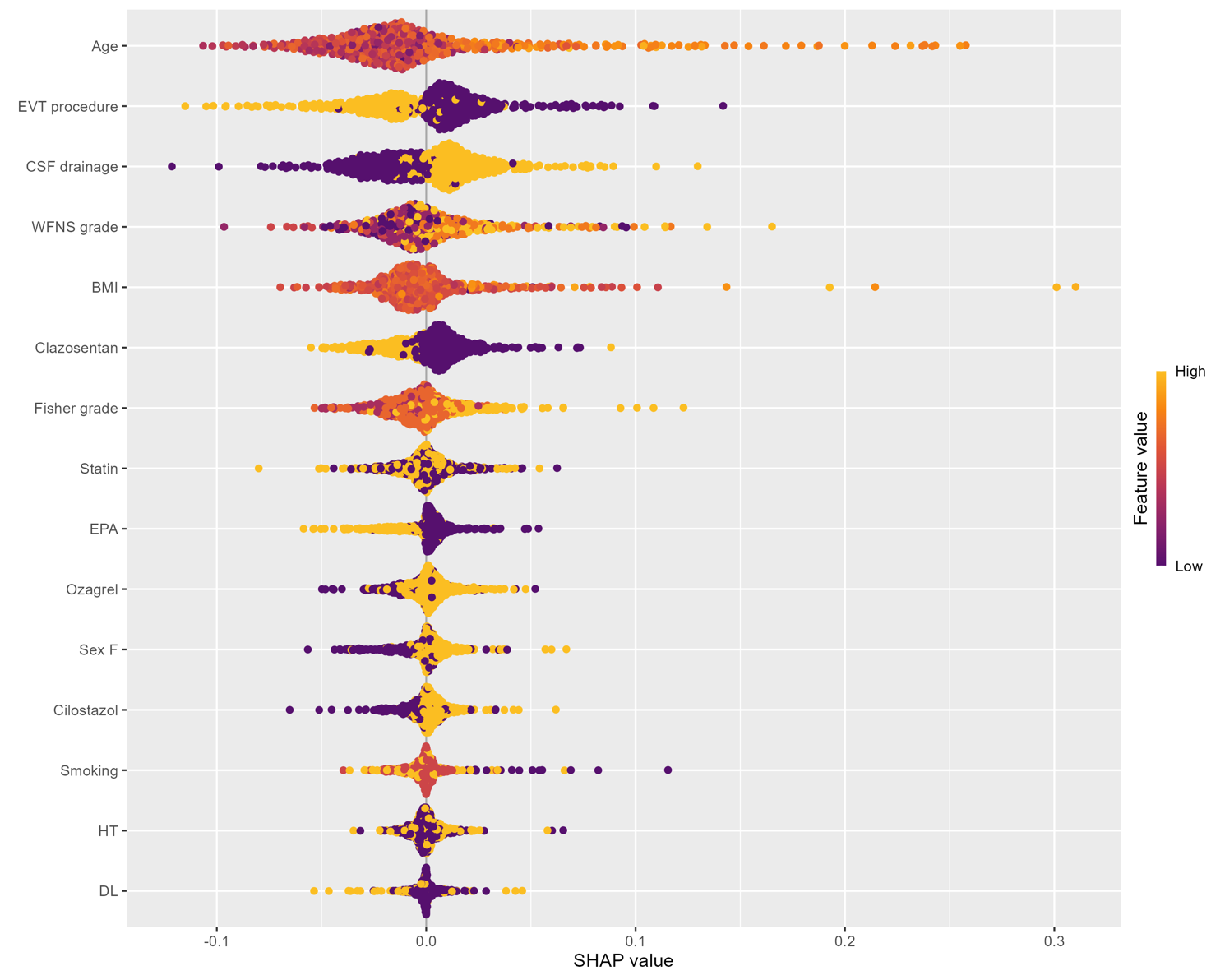


This figure visualizes the contribution of each feature to the predicted outcome (e.g., poor prognosis risk) in the machine learning model using SHAP (SHapley Additive exPlanations) values. The vertical axis lists the features included in the model, including age, EVT procedure, CSF drainage, WFNS grade, BMI, clazosentan, Fisher grade, statin use, EPA, ozagrel, female sex (Sex F), cilostazol, smoking, hypertension (HT), and dyslipidemia (DL). The horizontal axis represents the SHAP values, which indicate the magnitude and direction of each feature’s contribution to the predicted value. The color of the points represents the value of each feature, where purple indicates low feature values and yellow indicates high feature values.

A positive SHAP value (points to the right) indicates that the feature contributes to an increase in the predicted outcome (e.g., higher risk of poor prognosis), while a negative SHAP value (points to the left) indicates that the feature contributes to a decrease in the predicted outcome (e.g., lower risk of poor prognosis). Features are ordered on the vertical axis based on their overall contribution to the model. This provides a clear visual representation of both the magnitude and direction of each variable’s impact on the prediction, showing how high and low feature values influence the predicted outcomes.

**Supplementary Figure 4. Results of Multivariate Logistic Regression Analysis for Risk Factors Associated with Pulmonary Complications and Brain Edema (Forest Plot)**


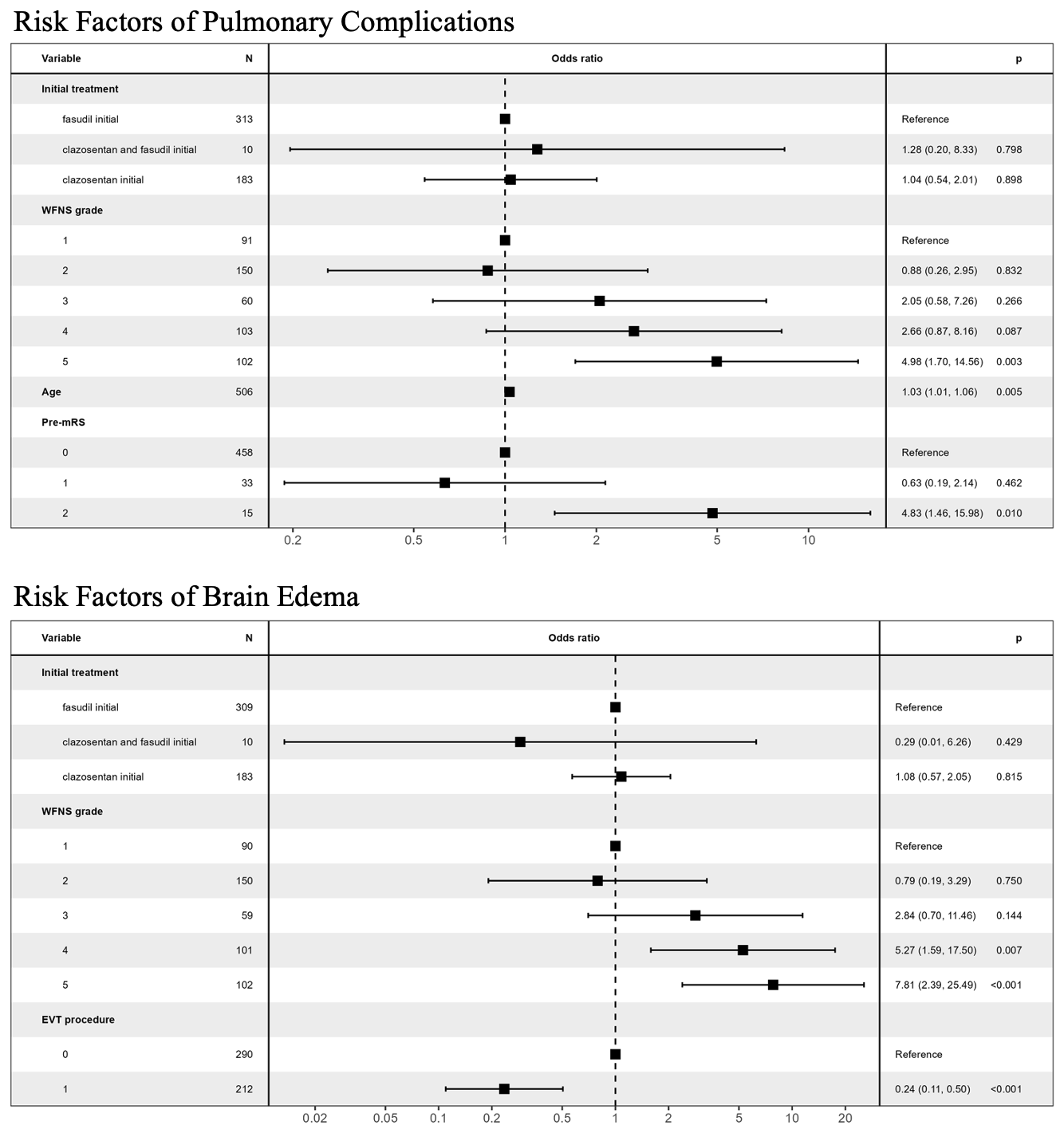


This figure presents the results of a multivariate logistic regression analysis evaluating risk factors for pulmonary complications (top panel) and brain edema (bottom panel) in patients with aneurysmal subarachnoid hemorrhage (aSAH) due to ruptured cerebral aneurysms. The horizontal axis represents the odds ratio (OR) on a logarithmic scale. The black squares indicate the point estimates of the OR, and the horizontal lines represent the 95% confidence intervals (95% CI). The vertical dashed line (OR=1) serves as the reference point, where values to the right indicate an increased risk, while those to the left indicate a decreased risk. P-values are displayed in the accompanying table, with p<0.05 considered statistically significant. In the top panel, assessing pulmonary complications, the explanatory variables included the type of initial treatment (reference: fasudil group), WFNS grade (reference: grade 1), age, and pre-mRS (modified Rankin Scale; reference: pre-mRS=0). The results indicate that WFNS grade 5, older age, and pre-mRS = 2 were significantly associated with an increased risk of pulmonary complications. In the bottom panel, presenting brain edema, the same explanatory variables were used, with the presence or absence of brain edema as the outcome. The results suggest that WFNS grades 4 and 5 were significantly associated with an increased risk of brain edema. Conversely, endovascular treatment (EVT procedure) was identified as a protective factor, significantly reducing the risk of brain edema.

**Supplementary Figure 5. Summary Plot Showing the Contribution of Each Variable (SHAP Value) in the Prediction Model: Pulmonary Complications**


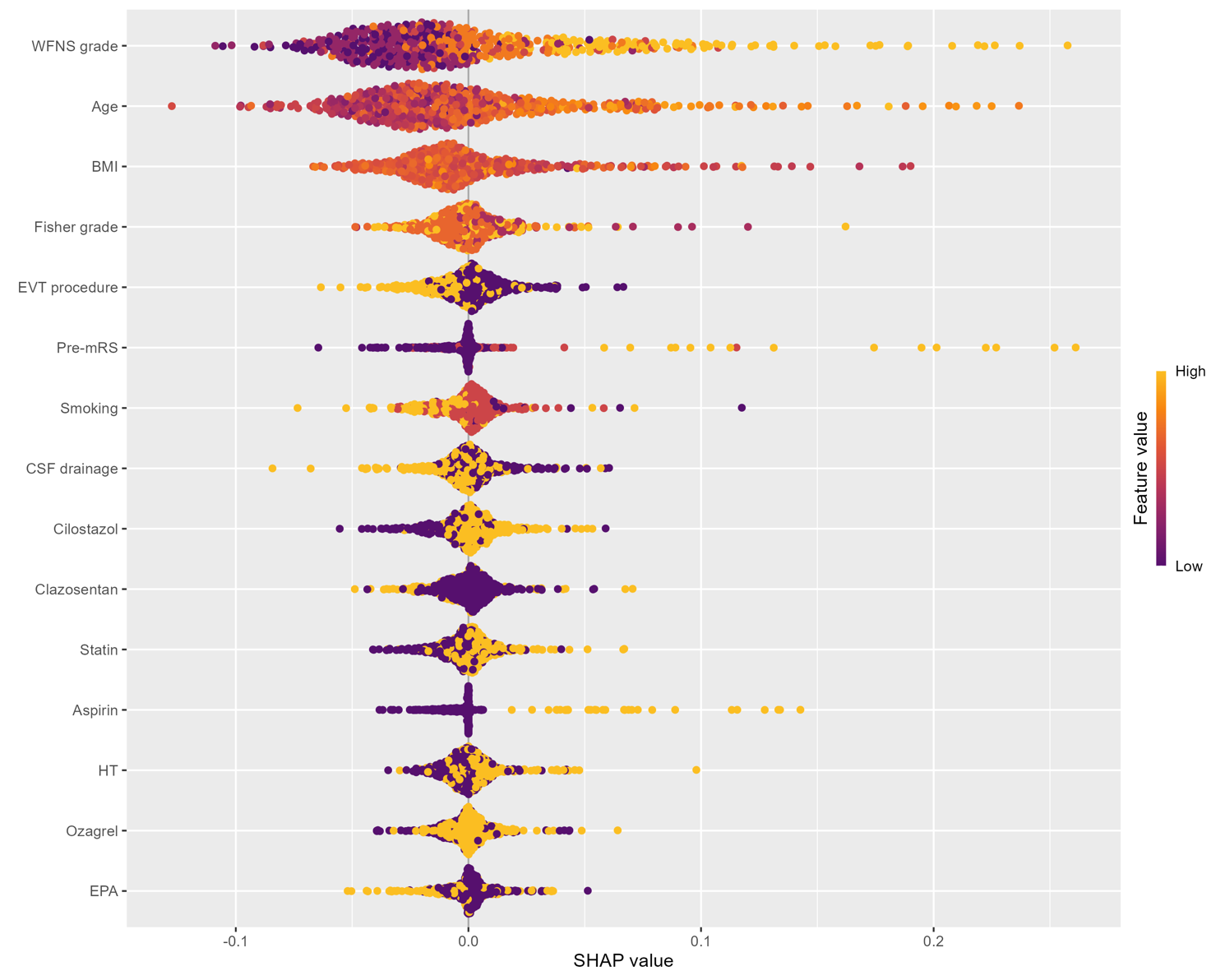


This figure presents a summary plot that visualizes the contribution of each feature to the prediction of outcomes in a machine learning model, using SHAP (SHapley Additive exPlanations) values.

The vertical axis lists the features included in the prediction model, including WFNS grade, age, Fisher grade, and the presence or absence of specific drug administrations. The horizontal axis represents the SHAP values, which indicate the magnitude and direction of each feature’s contribution to the predicted outcome. Each dot corresponds to an individual patient sample. The color of the dots reflects the magnitude of the feature values, where purple dots represent lower feature values and yellow dots represent higher feature values.

A positive SHAP value (dots to the right of the vertical dashed line, OR=1) indicates that the feature contributes to predicting a worse prognosis, while a negative SHAP value (dots to the left of the dashed line) indicates that the feature contributes to predicting a better prognosis. The further a dot is from the vertical dashed line, the greater the influence of that feature on the prediction. This summary plot provides an overview of the overall impact of each feature on the prediction model and highlights how high and low feature values affect the predicted outcomes.

**Supplementary Figure 6. Summary Plot Showing the Contribution of Each Variable (SHAP Value) in the Prediction Model: Brain Edema**


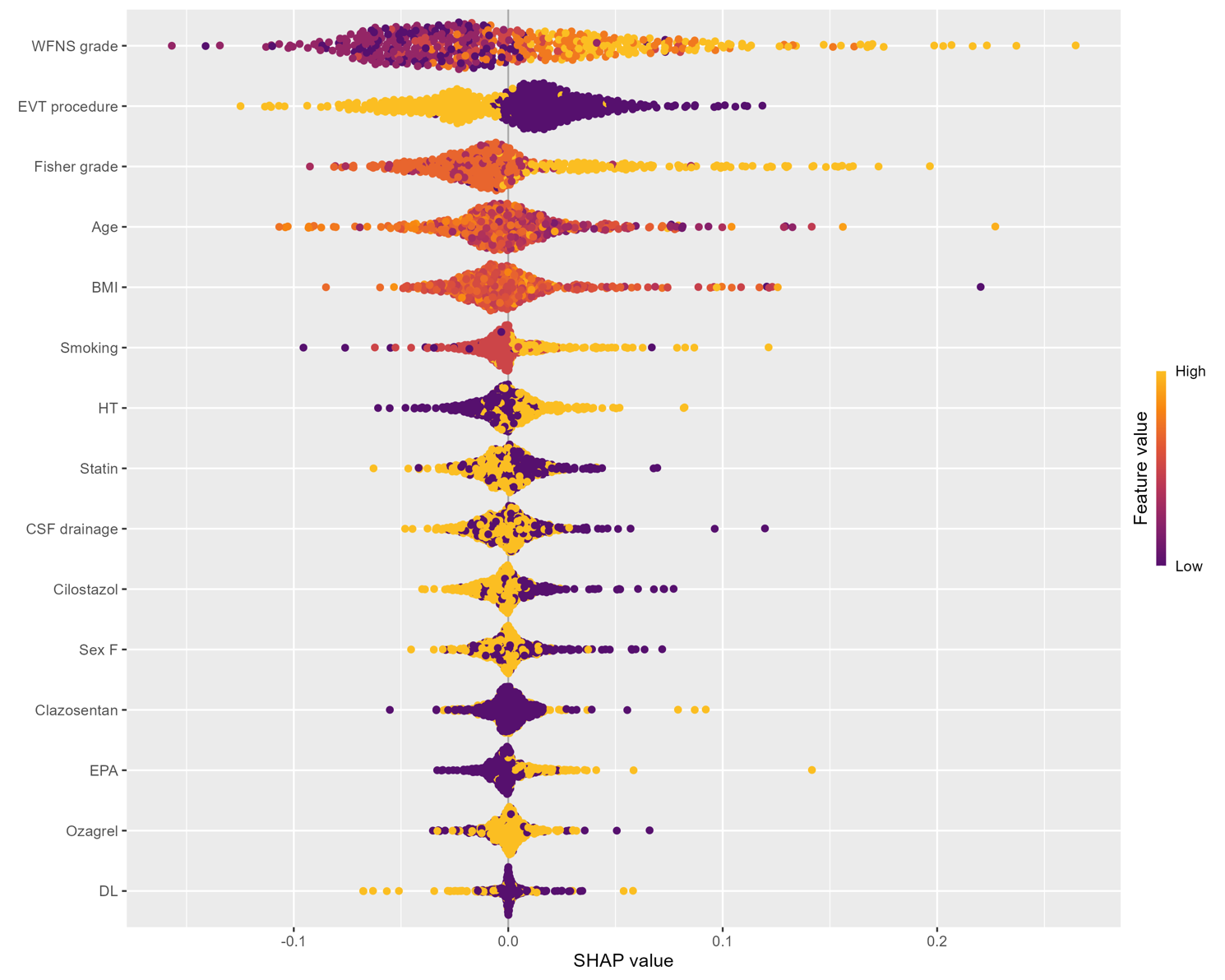


This figure is a summary plot that visualizes the contribution of each feature to the prediction of prognosis using SHAP (SHapley Additive exPlanations) values in a machine learning model. The vertical axis lists the features included in the prediction model, including WFNS grade, EVT procedure, Fisher grade, age, BMI, smoking, hypertension (HT), statin use, CSF drainage, cilostazol, female sex (Sex F), clazosentan, EPA, ozagrel, and dyslipidemia (DL). Each dot on the vertical axis represents an individual case. The horizontal axis represents the SHAP value, which indicates the magnitude and direction of each feature’s contribution to the predicted outcome. The color of the dots corresponds to the magnitude of the feature values, where purple dots represent lower feature values and yellow dots represent higher feature values.

A positive SHAP value (dots to the right of the vertical dashed line) indicates that the feature contributes to a higher predicted value (e.g., poor prognosis), while a negative SHAP value (dots to the left) indicates that the feature contributes to a lower predicted value (e.g., good prognosis). Features that have a greater overall importance to the model are placed higher on the vertical axis. This plot offers a clear visualization of how each feature influences the prediction, presenting both the magnitude and the direction of its effect on the outcome at different value levels.
